# A Case of Adult-Onset Atypical Teratoid/Rhabdoid Tumor With Extracranial Metastasis

**DOI:** 10.7759/cureus.81226

**Published:** 2025-03-26

**Authors:** Kengo Hirayama, Hirofumi Oyama, Kenichi Wakabayashi

**Affiliations:** 1 Department of Neurosurgery, Tosei General Hospital, Seto, JPN; 2 Department of Rehabilitation Medicine, Ogaki Municipal Hospital, Ogaki, JPN; 3 Department of Neurosurgery, Toyohashi Municipal Hospital, Toyohashi, JPN

**Keywords:** adult onset, at/rt, atypical teratoid/rhabdoid tumor, distant metastasis, sellar tumor

## Abstract

A 47-year-old woman visited a hospital with complaints of headache and epistaxis. A mass lesion was found in the sellar region, and the patient underwent partial removal via transsphenoidal surgery at the hospital. Pathological diagnosis revealed an atypical teratoid/rhabdoid tumor (AT/RT), and she was referred to our hospital for postoperative adjuvant therapy. Although chemotherapy with ifosfamide, carboplatin, and etoposide (ICE regimen) was administered, the residual tumor rapidly grew, and the patient presented with visual disturbance. Irradiation significantly reduced the tumor size. She was stable for 17 weeks; however, dissemination occurred in the left frontal lobe and lumbar spinal cord, and multiple metastases to the lungs occurred. The patient was treated with additional irradiation, oral temozolomide, intrathecal methotrexate, and intrathecal cytarabine; however, these treatments were ineffective. The patient died 34 weeks postoperatively. AT/RTs are rare malignant embryonal tumors that primarily occur in infants and children under three years of age. Due to its rarity, a standardized treatment has not been established. Adult-onset AT/RTs are extremely rare, with a limited number of reported cases. Extracranial metastases are also uncommon and have only been reported in a few cases. The accumulation of additional cases is necessary to establish a standardized treatment. Therefore, here, we report an adult case of extracranial metastasis to contribute to this effort.

## Introduction

Atypical teratoid/rhabdoid tumors (AT/RTs) are rare, accounting for 6.1% of malignant central nervous system tumors in children. These are highly malignant embryonal tumors, and 68.4% of them occur in infants and children under three years of age [1]. From a molecular genetic perspective, nearly all cases exhibit biallelic inactivation (deletion or mutation) of the INI1 (hSNF5, SMARCB1) gene located on chromosome 22q11.2, and this loss of function is considered the primary driver of tumor development. In extremely rare cases, mutations in SMARCA4/BRG1 have also been reported. Histologically, rhabdoid cells with eosinophilic cytoplasm and inclusion-like structures are characteristic. However, when these distinctive features are not well-developed, differentiation from medulloblastoma and other tumors can be challenging. Immunohistochemically, SMARCB1/INI1 staining is negative in tumor cells, which can lead to a diagnosis of AT/RT.

Currently, there is no established standard treatment. Depending on the case, multimodal therapies such as the Medical University of Vienna (MUV)-ATRT protocol or European Rhabdoid Registry (EU-RHAB) protocol are employed. However, the prognosis remains extremely poor, with a five-year survival rate of only 39.5% [1]. Adult-onset AT/RT is extremely uncommon, with a limited number of reported cases. Extracranial metastases of adult-onset AT/RT are even more uncommon, and only three cases of extracranial metastases have been documented. There is also no established standard treatment for adult cases, so the accumulation of additional cases is necessary. Herein, we report a case of adult-onset AT/RT, describe its clinical course, and discuss the characteristics based on a literature review.

## Case presentation

**Figure 1:**
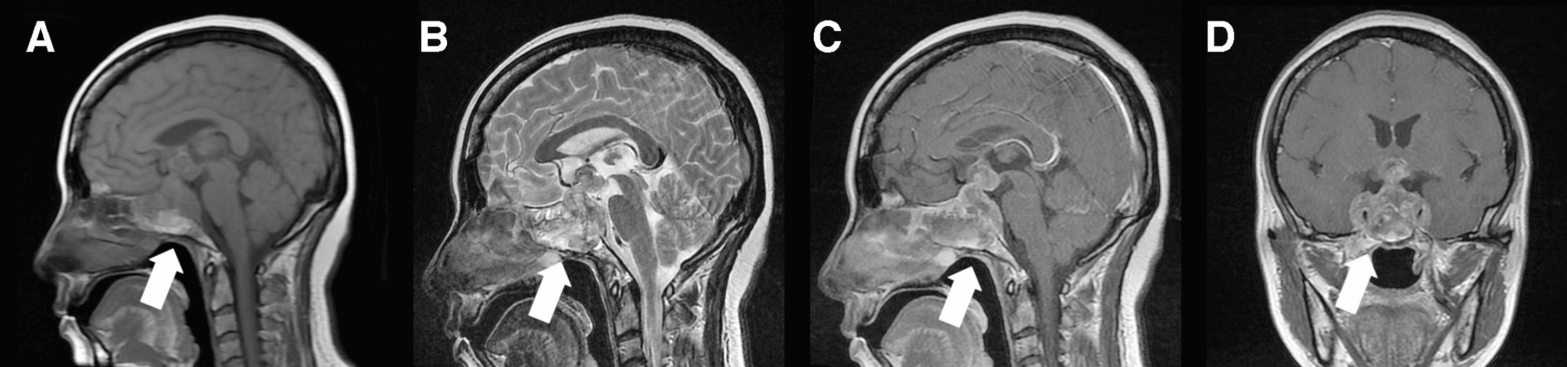
Preoperative MRI A and B: T1-weighted (A) and T2-weighted (B) sagittal MRI scans showing a mass lesion extending from the sphenoid sinus to the suprasellar region (arrows). C and D: Gadolinium-enhanced sagittal (C) and coronal (D) MRI scans showing increased mass enhancement with cavernous sinus invasion (arrows). MRI: magnetic resonance imaging

**Figure 2:**
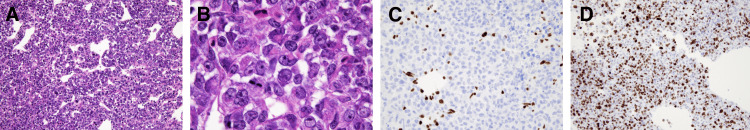
Histopathological Findings A and B: Hematoxylin and eosin staining showing scattered rhabdoid cells with acidophilic cytoplasm and inclusion body-like structures. C: Immunostaining revealing negativity for SMARCB1/INI1. D: MIB-1 index is 50%-60%.

**Figure 3:**
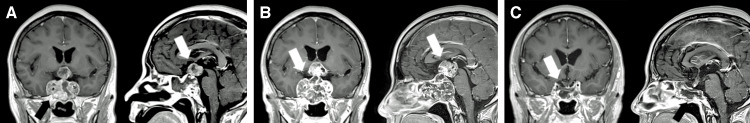
MRI During Chemotherapy and Radiation Therapy A: Gadolinium-enhanced MRI just before chemotherapy revealing regrowth of the residual tumor. B: Gadolinium-enhanced MRI 37 days after the induction of chemotherapy demonstrating rapid progression of the tumor. C: Gadolinium-enhanced MRI after radiation therapy showing a significant reduction in tumor size. MRI: magnetic resonance imaging

A 47-year-old woman visited a hospital complaining of a three-week progressive headache and epistaxis that she had experienced a week earlier. Additionally, she experienced irregular menstruation for one year. To date, no remarkable family history has been reported. Magnetic resonance imaging (MRI) revealed a 20 × 15 × 13 mm tumor extending from the sphenoid sinus to the suprasellar region (Figure 1). Laboratory data indicated an elevated prolactin level of 169 ng/mL and a low thyroid-stimulating hormone level of 0.036 μIU/mL (Table 1), whereas the levels of other pituitary hormones remained normal. The visual function also remained intact. The patient presented with diplopia due to right oculomotor and abducens nerve palsy. Three weeks later, she developed right facial numbness and left oculomotor nerve palsy. On the 23rd day after her first visit, the patient underwent partial tumor resection using the transsphenoidal approach. Histopathological examination revealed rhabdoid cells with an eosinophilic cytoplasm and inclusion-like structures. Moreover, immunohistochemical staining demonstrated the absence of SMARCB1/INI1 expression (Figure 2), confirming the diagnosis of AT/RT. The patient was referred to our institution on postoperative day 8 for adjuvant therapy. Upon admission, she was diagnosed with diabetes insipidus. Additional MRI revealed no evidence of spinal lesions, and cerebrospinal fluid cytology was negative. However, the rapid growth of the residual tumor in the suprasellar region (Figure 3A) led to progressive bilateral visual impairment.

**Table 1:** Preoperative Laboratory Data LDH: lactate dehydrogenase, GH: growth hormone, TSH: thyroid-stimulating hormone, ACTH: adrenocorticotropic hormone, LH: luteinizing hormone, FSH: follicle-stimulating hormone, ADH: antidiuretic hormone

Parameters	Unit and value	Reference range
Total protein	7.4 g/dL	6.2-8.0 g/dL
Total bilirubin	0.7 mg/dL	0.2-1.0 mg/dL
Urea nitrogen	6.4 mg/dL	7-20 mg/dL
Creatinine	0.61 mg/dL	0.6-1.2 mg/dL
LDH	240 U/L	115-245 U/L
C-reactive protein	2.31 mg/dL	0-0.5 mg/dL
Sodium	136 mEq/L	133-145 mEq/L
Potassium	3.9 mEq/L	3.3-5.0 mEq/L
Chloride	97 mEq/L	98-109 mEq/L
White blood cell count	4,900/µL	3,500-9,000/µL
Hemoglobin	14.7 g/dL	11.3-14.9 g/dL
Platelet count	226,000/µL	100,000-380,000/µL
GH	1.28 ng/mL	0.13-9.88 ng/mL
TSH	0.036 μIU/mL	0.541-4.261 μIU/mL
ACTH	6.8 pg/mL	7.2-63.3 pg/mL
LH	0.22 mIU/mL	0.5-15.0 mIU/mL
FSH	4.34 mIU/mL	0.5-5 mIU/mL
Prolactin	169 ng/mL	0-15 ng/mL
ADH	0.6 pg/mL	0-2.8 pg/mL

Chemotherapy with ifosfamide (1,900 mg/m^2^), carboplatin (400 mg/m^2^), and etoposide (130 mg/m^2^) (ICE regimen) was administered at this time. An MRI performed 10 days after the initiation of chemotherapy revealed tumor shrinkage, accompanied by improvement in symptoms. However, rapid tumor regrowth was observed 37 days after the initiation of chemotherapy (Figure 3B), resulting in bilateral visual impairment and right external ophthalmoplegia.

**Figure 4:**
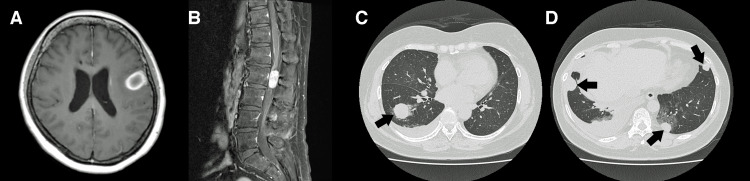
MRI and Computed Tomography Six Months After Initial Treatment A and B: Gadolinium-enhanced MRI six months after initial treatment demonstrating dissemination in the left frontal lobe and the lumbar spinal cord. C and D: Computed tomography six months after initial treatment revealing multiple lung metastases (arrows). MRI: magnetic resonance imaging

The second course of ICE chemotherapy (80% dose due to bone marrow suppression) was administered concurrently with irradiation (54 Gy/30 fractions) targeting the sphenoid sinus and suprasellar regions. Following radiotherapy, significant tumor shrinkage was noted (Figure 3C), and visual impairment improved. The third course of ICE chemotherapy (60% dose for the same reason) was administered. The patient remained progression-free for some time; however, 191 days after the initial chemotherapy, she developed dysarthria and pain in the right lower limb. MRI revealed dissemination to the left frontal lobe and lumbar spinal cord (Figures 4A, 4B); meanwhile, whole-body computed tomography confirmed the presence of multiple pulmonary metastases (Figures 4C, 4D). Radiotherapy was administered to the left frontal lobe (35 Gy/5 fractions) and lumbar lesions (39 Gy/13 fractions), along with oral temozolomide (90 mg/m²) and intrathecal delivery of methotrexate (9 mg/m²) and cytarabine (30 mg/m²). Radiotherapy was also administered to the rapidly enlarging lesion in the right lower lobe of the lung but was discontinued at 33 Gy/11 fractions due to worsening pleural effusion. Subsequently, tumor progression could not be controlled. The patient died 254 days (36 weeks) after the introduction of the ICE regimen.

## Discussion

An AT/RT is a rare and highly malignant embryonal tumor. The predominant age of onset is under three years [1]. In contrast, adult cases accounted for approximately 2% of all cases. Due to the rarity of the tumor, a standard treatment, even for pediatric cases, has not yet been established. Moreover, various chemotherapy regimens using agents such as cisplatin, carboplatin, cyclophosphamide, etoposide, and vincristine, as well as radiation therapy, are utilized. In adults, knowledge and evidence are even more scarce.

We reviewed the literature on adult-onset AT/RTs and identified 80 cases in 48 articles [2-18]. Clinical information, including age, treatment details, and prognosis, was available for 73 of these cases, which we analyzed. The details are summarized in Table 2. The median age of adult-onset AT/RTs was 38 years (range: 15-73 years), with a slight predominance in females (64.4%, n=47). The most common tumor location was the sellar region (46.6%, n=34), followed by the pineal region (16.4%, n=12), cerebral hemisphere (13.7%, n=10), posterior fossa (11.0%, n=8), and spinal canal (6.8%, n=5). Tumors in the sellar region and spinal canal were more common in females (85% and 80%, respectively), whereas those in the cerebral hemisphere were predominantly identified in males (70%). Our case was typical of adult-onset AT/RT in terms of age at onset, sex, and location. The extent of resection was documented in 52 cases in the literature. Gross total resection, subtotal or partial resection, and biopsy were performed in 19 cases (26%), 30 cases (41.1%), and three cases (4.1%), respectively.

**Table 2:** Characteristics of Patients With Adult-Onset Atypical Teratoid/Rhabdoid Tumor in the Literature

Parameters	Number (%)		
Number of cases	73		
Age, median (range)	38 (15-73)		
Sex	Male	Female	
	26 (35.6%)	47 (64.4%)	
Location	Total	Male	Female
Sellar	34 (46.6%)	5 (14.7%)	29 (85.3%)
Pineal	12 (16.4%)	6 (50.0%)	6 (50.0%)
Hemisphere	10 (13.7%)	7 (70.0%)	3 (30.0%)
Posterior fossa	8 (11.0%)	3 (37.5%)	5 (62.5%)
Spinal	5 (6.8%)	1 (20.0%)	4 (80.0%)
Others	4 (5.5%)	4 (100.0%)	0 (0.0%)
Extent of resection			
Gross total resection	19 (26.0%)		
Subtotal or partial resection	30 (41.1%)		
Biopsy	3 (4.1%)		
Not described	21 (28.8%)		
Median survival time		Months	
Overall	73 (100%)	24	
Surgery alone	14 (19.2%)	2.5	
Surgery + chemoradiotherapy	32 (43.8%)	33	
Surgery + chemotherapy	3 (4.1%)	13	
Surgery + radiotherapy	16 (21.9%)	20	
Adjuvant therapy: details unknown	8 (11.0%)	6	
Chemotherapeutic agents			
Etoposide	21 (28.8%)		
Cisplatin	16 (21.9%)		
Ifosfamide	14 (19.2%)		
Vincristine	12 (16.4%)		
Cyclophosphamide	11 (15.1%)		
Carboplatin	9 (12.3%)		
Temozolomide	9 (12.3%)		
Others	13 (17.8%)		

The median survival time of the 73 patients was 24 months. Among the post-surgical treatment modalities, excluding eight cases in which the administration of chemotherapy or radiation therapy was unclear in the literature, patients receiving combined chemoradiotherapy (43.8%, n=32) had a relatively long median survival of 33 months, whereas those receiving chemotherapy (4.1%, n=3) or radiation therapy (21.9%, n=16) alone had median survivals of 13 and 20 months, respectively. The most frequently administered chemotherapy regimens included etoposide (28.8%, n=21) or cisplatin (21.9%, n=16). Intrathecal chemotherapy was administered in six patients, and autologous stem cell transplantation (ASCT) was performed in five patients. A cohort study of pediatric AT/RT patients treated with high-dose chemotherapy and ASCT reported a five-year overall survival rate of 100% and a five-year progression-free survival rate of 88.9% [19]. In adults, a case report using ASCT described a 17-month disease-free rate [15]. Therefore, ASCT could be an effective treatment option. In pediatric cases, the effectiveness of Aurora kinase inhibitors and EZH2 inhibitors have been reported. The accumulation of pediatric cases and the potential application of these therapies to adult patients are anticipated.

Our patient was managed primarily with ICE chemotherapy, a regimen employed for pediatric medulloblastoma, due to concerns regarding spinal dissemination. However, ICE alone was insufficient for tumor control, and the addition of radiation therapy resulted in a relatively stable period. Previous reports on pediatric cases have widely acknowledged the effectiveness of radiotherapy. Additionally, early introduction of radiotherapy has been associated with improved survival outcomes [20]. However, further research is needed to determine the optimal timing of radiotherapy. In our case, oral temozolomide and intrathecal administration of methotrexate and cytarabine, which are included in some pediatric treatment protocols, were performed for tumor recurrence and spinal metastasis. However, its effectiveness was limited. The efficacy of intrathecal administration has not been established, and further research is also needed.

Our patient developed extracranial metastases, a phenomenon rarely reported in adult patients with AT/RT. To date, three cases of extracranial metastases have been documented in adult patients with AT/RT (Table 3). All cases, including ours, originated in the sellar region. Three cases developed metastases to the lungs (one of which also had metastasis to the lumbar spinous process), and one case involved metastasis to the peripheral nerves. All patients, except for one with unknown details, underwent partial resection. The period from diagnosis to metastasis ranged from three months to 10 years. Long-term survival after the detection of metastasis was achieved in only one case (25 months), while the remaining three cases resulted in death within three months, indicating an extremely poor prognosis. Sellar lesions suggest the potential for hematogenous metastasis due to cavernous sinus invasion [6]. For patients with partially resected sellar lesions, close monitoring is essential for identifying cases of extracranial metastases.

**Table 3:** Reported Cases of Extracranial Metastasis in Adult Atypical Teratoid/Rhabdoid Tumor CT: chemotherapy, HD-CT: high-dose chemotherapy, RT: radiation therapy, NA: not available

Authors, year	Age/sex	Location	Initial treatment	Time from surgery to metastasis	Metastasis	Treatment after metastasis	Outcome
Moretti et al. (2013) [14]	60/female	Sellar	Surgery alone	5 months	Lung	RT, CT	Death after 30 months
Johann et al. (2019) [8]	20/female	Sellar	Surgery, HD-CT	120 months	Peripheral nerve	NA	Death after 120 months
Fukuda et al. (2022) [6]	45/female	Sellar	Surgery, RT, CT	NA	Lung, L4-5 spinous process	RT, CT	Death after 5 months
Present case	47/female	Sellar	Surgery, RT, CT	29 weeks	Lung	RT, CT	Death after 8 months

AT/RT is highly challenging to treat in both pediatric and adult cases. The treatment methods for adult AT/RT vary significantly across the literature, and there is no standardized protocol, making it difficult to evaluate detailed clinical courses and treatment efficacy. Further accumulation of these cases is essential, and prospective data collection, collaborative registries, and efforts to standardize treatment strategies are considered to elucidate their pathophysiology and establish effective treatments.

## Conclusions

Here, we report a case of adult-onset AT/RT with extracranial metastases. Radiation therapy was deemed to be relatively effective. Partial resection of sellar lesions is likely to result in extracranial metastasis. Further accumulation of adult-onset AT/RT cases is essential, and prospective data collection and collaborative registries are considered to elucidate their pathophysiology and establish effective treatments.
